# Reply of Medicus to Dr. Gregory

**Published:** 1828-02

**Authors:** 


SMALL-POX.

*Reply of* Medicus *to* Dr. Gregory.

I have just read, in your Number for this month, Dr. Gregory's reply to what he is pleased to call my "animadversions" on his paper on Constitutional Inaptitude to Cow-Pox. As he is evidently somewhat out of temper with them, it does not appear to me, who am a man of peace, quite safe that I should at present expose myself to his critical wrath. To you, however, who so readily gave a place to my former observations, some acknowledgment, and a few additional remarks, are due. Should you think these worthy of the attention of your readers, they are at your and their service: but I deem it prudent, for this time at least, to preserve my incognito.

I shall not descend into the arena to "chop logic" with the learned Doctor; yet I may observe, *en passant*, that if there be, as he admits there is, a constitutional insusceptibility of small-pox,—*small-pox* which Dr. Jenner held to be the virulent and *contagious* form of the disease, my argument, *a fortiori*, was neither illogically nor irrationally applied to infer a like unsusceptibility of cow-pox, the mild and *non-contagious* variety. With this avowed view of the subject, I could not possibly "mean to say *pari ratione*," especially as I wished to speak intelligible language." I cannot pretend to follow out the Doctor in his "case reversed," nor yet his *illustration* of walking twenty miles and ten, lest, in the "palpable obscure" to which they lead, I might not merely "stumble" but stray; and here I would whisper gently in his ear, "Cave ne titubes mandataque frangas." This, I hope, he will take in friendly part, as an *argumentum ad hominem*.

If he be determined not to avail himself of his own experience, nor yet that of others who have gone before him, "Baron Dimsdale and the inoculators of the old school," he has certainly no fair claim on me for the information he requires; more particularly as he seems to impute to me the crime of being a young and unfledged "practitioner." To that heavy charge, at least, I can truly plead not guilty; and have only further to say of myself, that, whatever may be my other disqualifications, I trust I shall not be found among the number of those who, as they recede from youth, advance in obstinacy, and grow old in ignorance in spite of experience.

I do not mean to be driven by the multiplicity or variety of Dr. Gregory's questions from what I take to be the real point at issue. My short and hasty letter was called forth by Dr. Gregory's paper, which sought information respecting Dr. Jenner's opinions on a subject that I knew had been very fully and satisfactorily treated in his "Life." Dr. G.'s first questions, one and all, resolve themselves into a knowledge of the laws which regulate variola and variolæ vaccinæ. As I was well aware that Dr. Jenner's opinions on these topics were set forth in that work; and as I believed that Dr. Gregory had either not read it, or had examined its contents in a very cursory way, I referred him to three chapters. In these chapters I see the pathological phenomena illustrated in a very striking manner by the literary history; I see a great number of facts, which heretofore appeared discordant and inexplicable, elucidated with irrestible force; and all the plain, practical deductions of Jenner confirmed with a clearness of evidence which I have no doubt will ultimately be sufficient to put an end to all the blunders both in the theory and practice of vaccination which have thus far unfortunately prevailed.

Let Dr. Gregory read again the chapters already referred to, and see if he can find out any statements that are not supported by evidence, and have not a direct practical tendency. As he seems not yet to have made this examination, I will tell him what he may find in these chapters. In the first place, sir, he will discover that an eruptive disease, of a variolous nature, has been known to exist in man, and several of the inferior animals, from the earliest periods of authentic history. In the next place, it is proved that the epizootic disease, most clearly described as variola by competent witnesses, has been traced in England and in other parts of the world. In the third place, it has been rendered more than probable that the variolæ vaccinæ, as discovered by Jenner, are the local remains of the more violent epidemic. It is

6

impossible here to bring forward all the proofs by which these points are sustained, but I do contend that they are made out with a degree of certainty that may fairly defy all attempts to gainsay them.

If Dr. Gregory demands "chapter and verse," let him look to pages 163 et seq. usque ad 206; then again to 237-239, 240, 241, 352. Indeed, nothing is more easy than to multiply references to almost every page of the three chapters mentioned. Now let us examine how these historical facts apply to the leading points in question. Dr. Jenner found an eruptive disease of the cows in Gloucestershire. This disease, when communicated to man, afforded protection from small-pox. From an attentive examination of its characters, he was led to believe that it was a mild variety of the small-pox, and he accordingly called it Variolæ Vaccinæ. The whole of his subsequent experience confirmed this opinion, and he contended that the same general laws which governed the variolæ were applicable to the variolæ vaccinæ. These doctrines, derived from attentive observation, are stated in the clearest and most emphatic manner in Dr. Baron's book.

But Dr. Gregory will most likely say, "my original questions are still unanswered." Speaking literally, it may be so; but logically and by direct inference, they assuredly are. The pathological facts led Dr. Jenner to the conclusions which we have seen. The historical evidence confirms these conclusions, and moreover throws a flood of light on the origin of cow-pox. Now, all this proves, first, that Dr. Jenner had accurately traced the laws of small-pox and of cow-pox; and, secondly, that his opinions have received a most striking confirmation from events which occurred anterior to his discovery, as well as those which have presented themselves since.

Here let us take another view of this matter. What was the object of Dr. Baron in undertaking the illustration of Dr. Jenner's opinions? Was it not to do the very thing which Dr. Gregory contends has been left undone? Did he not collect many facts which bore upon the literary as well as medical history of small-pox and of cow-pox? Did he not show that the peculiarities of each affection are best elucidated by combining the examination of their pathological character with their literary history? (See his Introduction, page 14.) Did he not prove that Dr. Jenner, in investigating the properties of the variolæ vaccinæ, was guided by his knowledge of the laws which regulate small-pox? Did he not also prove that difficulties, which at first sight appeared insurmountable, were overcome by this very knowledge? (See pages 132, 133.) Did he not make it manifest, on a subsequent occasion, that the same knowledge enabled Dr. Jenner to explain the occasional causes of insecurity in the practice of vaccination? and that, as small-pox was known to have attacked the same individual twice, in like manner it might be expected at times to succeed cow-pox? (See page 278.) Did not all Dr. Jenner's writings, from the year 1799 downwards, show that he had anticipated the very questions which have now been put? Did he not, particularly in his second and third Treatises, endeavour to call the attention of his readers to some physiological facts respecting the nature of the small-pox infection, because he foresaw that they would eventually become connected with vaccination? Have not all these things been clearly announced in the Life of Dr. Jenner, and in his own published works? and will Dr. Gregory still say that he can find nothing that bears upon his first questions? Will he still say that Dr. Baron's views of the subject are speculative, and not strictly practical. For my own part, I, who am a practical man, can see nothing but what is practical in the beginning, in the middle, and in the end. The laws of the vaccine disease were deduced by Dr. Jenner from analogies drawn from the history of small-pox. The deviations in the former were illustrated by a reference to the same source, and many of the essential rules for its practice were derived therefrom. Was it a business of mere speculation to render all this manifest; and has it not all been incontestibly proved in Dr. B.'s book? Is not the illustration of the nature of small-pox and cow-pox the prominent object of Dr. Jenner and of his biographer throughout? As to the "constitutional inaptitude" to either of these affections, what would have been Dr. Jenner's answers to such questions as are put by Dr. Gregory? I have no doubt, both from his published writings and from the facts mentioned in his Life, that he would have replied to the following purport:—"My opinions respecting the variolæ vaccinæ were partly adopted, and ultimately confirmed, by my knowledge of the history of small-pox. I believe that both diseases are essentially the same. I know that small-pox infection will not render the constitution totally unsusceptible of that disease, and my opinion is that cow-pox, in the same way, may fail to protect. I likewise know that some constitutions resist small-pox contagion for many years, and afterwards imbibe it; others resist it altogether. The same thing is true with regard to cow-pox; and I have always considered both affections as governed by the same laws. For these opinions Dr.

Gregory may perhaps still demand "chapter and verse." If he does, he has only to read Dr. Jenner's publications, and to consult the passages already pointed out.

As to those individuals who enjoy "the inaptitude" to receive small-pox infection, it is manifestly impossible to calculate, either *a priori* or *a posteriori*, the ratio or the numbers, inasmuch as the same person has been well known to resist the infection even to a very late period of life, and afterwards to have had the disease in a malignant, nay even in a fatal, form.

It would, Mr. Editor, be happy for mankind if this "inaptitude," which now occupies Dr. Gregory's attention, were much more common than it is. In that case small-pox would be divested of many of its terrors, and Dr. Jenner's anxious and laborious investigations respecting its milder variety, the variolæ vaccinæ, would lose a great portion of their value. As to the inaptitude itself, it is manifestly an idiosyncracy, which has been long observed, and which is evidently incapable of being ascertained by any experience, whether individual or collective; because, as has been already remarked, it may and does exist at one period of life, and not at another. In some it possibly may continue through the whole of their lives; but of this we can never have full evidence previous to the close of each individual life.

MEDICUS.

*January 5th*, 1828.

---

